# Prognostic impact of CD73 and A2A adenosine receptor expression in non-small-cell lung cancer

**DOI:** 10.18632/oncotarget.14434

**Published:** 2017-01-02

**Authors:** Yusuke Inoue, Katsuhiro Yoshimura, Nobuya Kurabe, Tomoaki Kahyo, Akikazu Kawase, Masayuki Tanahashi, Hiroshi Ogawa, Naoki Inui, Kazuhito Funai, Kazuya Shinmura, Hiroshi Niwa, Takafumi Suda, Haruhiko Sugimura

**Affiliations:** ^1^ Department of Tumor Pathology, Hamamatsu University School of Medicine, Hamamatsu, Shizuoka, Japan; ^2^ Second Division, Department of Internal Medicine, Hamamatsu University School of Medicine, Hamamatsu, Shizuoka, Japan; ^3^ First Department of Surgery, Hamamatsu University School of Medicine, Hamamatsu, Shizuoka, Japan; ^4^ Division of Thoracic Surgery, Respiratory Disease Center, Seirei Mikatahara General Hospital, Hamamatsu, Shizuoka, Japan; ^5^ Department of Pathology, Seirei Mikatahara General Hospital, Hamamatsu, Shizuoka, Japan; ^6^ Department of Clinical Pharmacology and Therapeutics, Hamamatsu University School of Medicine, Hamamatsu, Shizuoka, Japan

**Keywords:** CD73, A2AR, adenosine, prognosis, non-small-cell lung cancer

## Abstract

In immune cells, CD73 dephosphorylates and converts extracellular AMP into adenosine, which binds the A2A adenosine receptor (A2AR). Blockade of this interaction, which induces an immunosuppressed niche in the tumor microenvironment, represents a potential novel treatment strategy. The clinical significance of CD73 and A2AR expression in non-small-cell lung cancer (NSCLC), however, has yet to be thoroughly investigated. Here we evaluated CD73 and A2AR protein expression levels using immunohistochemistry in tissue microarrays containing 642 resected NSCLC specimens. Furthermore, we compared the expression profiles of 133 paired primary tumors and lymph node metastases. CD73 and A2AR expression levels were significantly higher in females than in males, in never smokers than in ever smokers, and in adenocarcinomas than in squamous cell carcinomas. Among adenocarcinomas, significantly higher CD73 and A2AR expression was observed in TTF-1-positive and mutant EGFR-positive tumors than in their counterparts. Compared with CD73, A2AR expression was more inconsistent between primary tumors and lymph node metastases. Among NSCLC patients, high CD73 expression was an independent indicator of poor prognosis in multivariate Cox regression analyses for overall survival [hazard ratio (HR), 2.18; 95% confidence interval (CI), 1.38–3.46] and recurrence-free survival (HR, 2.05; 95% CI, 1.42–2.95). In contrast, high A2AR expression was an independent predictor of favorable prognosis for overall survival (HR, 0.70; 95% CI, 0.50–0.98) and recurrence-free survival (HR, 0.74; 95% CI, 0.56–0.97). Together, these findings indicate that CD73 and A2AR have opposing prognostic effects, although cases involving CD73 or A2AR expression share some clinicopathological features.

## INTRODUCTION

Despite significant advances in diagnosis, treatment, and care, the prognosis of non-small-cell lung cancer (NSCLC) has not improved satisfactorily. Further improvements in prognosis are needed even among patients who undergo surgical treatment as local recurrences and distant metastases occur frequently. New predictors of the outcomes of NSCLC are required to improve cancer management.

Immunosuppression within the tumor micro-environment has become an important and promising treatment target. Currently, the clinical applications of immune checkpoint inhibitors such as antibodies against programmed death-1 (PD-1), programmed death ligand-1 (PD-L1), and cytotoxic T lymphocyte antigen-4 (CTLA-4) have yielded significant antitumor activity and some durable responses in several malignancies, including NSCLC [1–5]. However, only a limited subset of patients with NSCLC received benefits from such therapy, and therefore more effective treatment strategies such as combinational immunotherapy are needed to improve outcomes to the extent observed in melanoma patients [5].

The cancer-derived adenosine pathway is an additional potential treatment target. CD73, also known as ecto-5’-nucleotidase, is a pivotal molecule that enzymatically dephosphorylates and converts extracellular adenosine monophosphate (AMP) into adenosine and inorganic phosphate [6]. Released extracellular adenosine binds to four specific G-protein-coupled adenosine receptors, A1, A2A, A2B, and A3 [7], among which the A2A adenosine receptor (A2AR) is the predominant form expressed in most immune cells [8] and is also expressed in lung cancer cells [9]. Extracellular adenosine generated by this pathway inhibits the functions of antitumor T cells and promotes T cell apoptosis, leading to immune escape by the tumor [10–12]. Independently of its immunosuppressive function, CD73 is also linked to the proliferation, migration, neovascularization, metastasis, and chemoresistance of tumor cells [11, 13–16]. Regarding clinical significance, tumor CD73 expression has been reported to associate with poor prognosis in several types of cancer, including colorectal [17], gastric [18], gallbladder [19], serous ovarian [20], and triple-negative breast cancers [21]. Conflicting effects of CD73 expression on survival, however, have also been reported [22, 23]. In addition, the expression profiles and clinical relevance of CD73 in NSCLC remain unknown. Moreover, little information is available about the prognostic effect of tumor A2AR expression and its association with the clinicopathological characteristics.

In the present study, we evaluated the CD73 and A2AR expression statuses of surgically resected tumor specimens from a sufficient number of patients with NSCLC. Associations between their expression profiles and clinicopathological characteristics, as well as patients’ prognoses, were analyzed.

## RESULTS

### CD73 and A2AR protein expression in NSCLC

**Figure 1:**
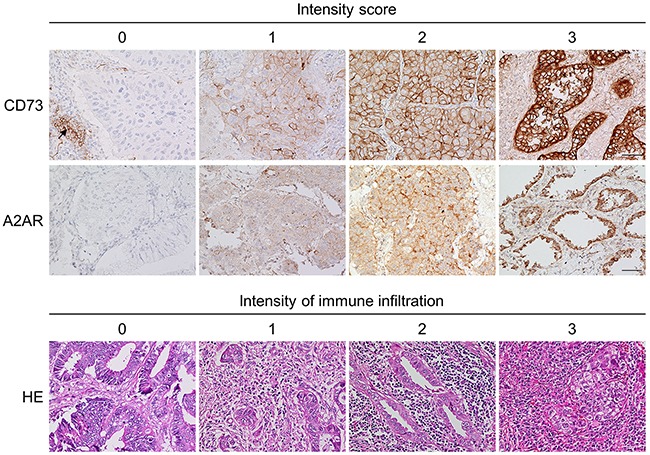
Representative images from an immunohistochemistry analysis of CD73 and A2AR and representative images for immune infiltration assessment Images of each CD73 membranous intensity level [0 (absent), 1 (weak), 2 (moderate), and 3 (strong), upper panel] and each A2AR membranous and cytoplasmic intensity level [0 (absent), 1 (weak), 2 (moderate), and 3 (strong), middle panel] are shown (original magnification, ×40). In the hematoxylin and eosin (HE)-stained panel (lower panel), images of each intensity of immune infiltration [0 (no), 1 (mild), 2 (moderate), and 3 (strong)] are shown (original magnification, ×40). The black arrow in the CD73-stained panel indicates CD73-positive lymphocytes, which are used as positive internal controls. The scale bars are 50 μm.

**Figure 2:**
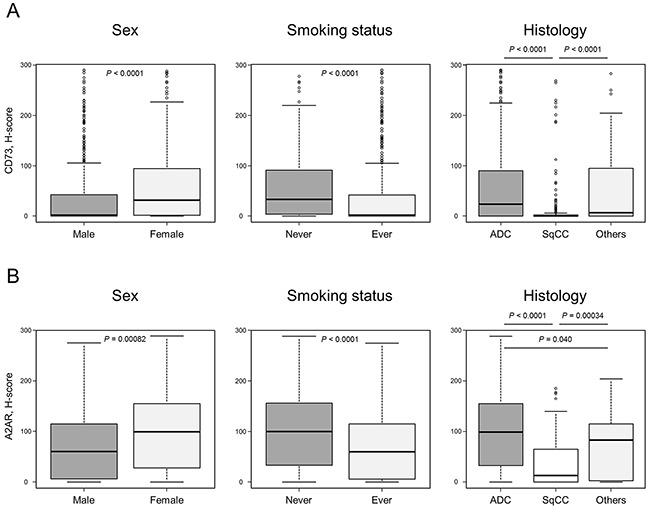
CD73 and A2AR expression levels according to clinicopathological factors CD73 **A.** and A2AR **B.** expression levels are plotted according to sex, smoking status, and histology. Each box plot indicates the median and interquartile range (top and bottom borders of the box). The whiskers above and below each box represent 1.5× of the interquartile range. Abbreviations: ADC, adenocarcinoma; SqCC, squamous cell carcinoma.

The patient characteristics included in this study are shown in Table 1. Among the 653 collected specimens, 11 were excluded from this study because of core loss or lack of a sufficient number of tumor cells following use in other research. Regarding patients in this study, 68.5% were male, 68.2% were ever smokers, and 65.7% and 27.3% had adenocarcinoma and squamous cell carcinoma, respectively. Representative examples of different CD73 and A2AR expression intensities are shown in Figure 1. The median CD73 and A2AR H-score values were 8 (interquartile range [IQR], 0–60; range, 0–290) and 75 (IQR, 8–133; range, 0–288), respectively. Significantly higher expression of both CD73 and A2AR was observed in women than in men, and in never smokers than in ever smokers (Figure 2). In addition, CD73 expression was significantly higher in adenocarcinoma and in other histologies than in squamous cell carcinoma (Figure 2). A2AR expression levels were significantly higher in adenocarcinoma than in squamous cell carcinoma or other histologies, and significantly lower in squamous cell carcinoma than in other histologies (Figure 2). Despite these shared features, no significant association was observed between CD73 and A2AR positivity (Fisher's exact test, *P* = 0.19). Furthermore, A2AR expression was significantly lower in tumors with high immune infiltration (*N* = 73, 11.4%) than in those with low immune infiltration (*N* = 569, 88.6%, *P* = 0.0052), whereas there was no significant difference in CD73 expression according to the intensity of immune cell infiltration (Supplementary Figure 1A).

**Table 1:** Clinicopathological characteristics of patients

Characteristic	Total(*N* = 642)*N* (%)
**Age (years)**	
median (range)	68 (23–88)
**Sex**	
Male	440 (68.5)
Female	202 (31.5)
**Smoking status**	
Never	191 (29.8)
Ever	438 (68.2)
Unknown	13 (2.0)
**Histology**	
Adenocarcinoma	422 (65.7)
Squamous cell carcinoma	175 (27.3)
Others ^a^	45 (7.0)
**p-T**	
1	264 (41.1)
2	279 (43.4)
3	62 (9.7)
4	37 (5.8)
**p-N**	
0	479 (74.6)
1	72 (11.2)
2	84 (13.1)
3	7 (1.1)
**Pathological stage**	
I	413 (64.3)
II	107 (16.7)
III	122 (19.0)
**Neoadjuvant chemotherapy**	
Yes	25 (3.9)
No	617 (96.1)
**Adjuvant chemotherapy**	
Yes	263 (41.0)
No	379 (59.0)

^a^ Others in histology include adenosquamous carcinoma (*N* = 21), large cell neuroendocrine carcinoma (*N* = 9), pleomorphic carcinoma (*N* = 7), large cell carcinoma (*N* = 5), giant cell carcinoma (*N* = 2), and carcinosarcoma (*N* = 1).

### Analysis of survival and CD73 and A2AR expression in NSCLC

**Figure 3:**
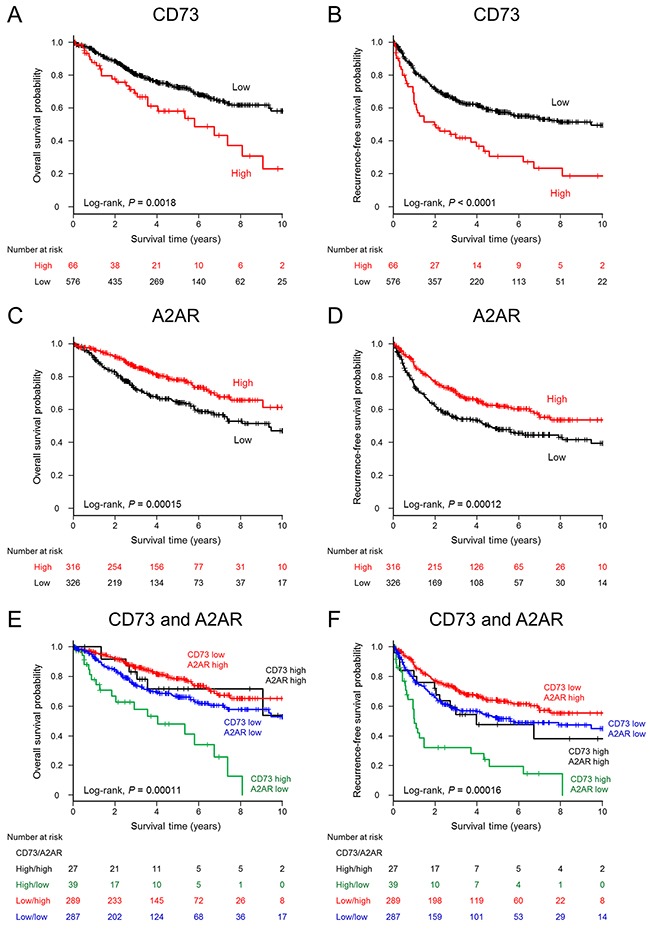
Kaplan–Meier estimates of overall survival and recurrence-free survival (years) stratified according to CD73 or A2AR expression levels in patients with non-small cell lung cancer Significant differences in overall survival **A.** and recurrence-free survival **B.** are shown for non-small-cell lung cancer (NSCLC) patients with high CD73 expression vs. those with low CD73 expression. Patients with NSCLCs harboring high A2AR expression had significantly better overall survival **C.** and recurrence-free survival **D.** outcomes than those with low A2AR expression. Survival estimations stratified by combined tumor CD73 and A2AR expression status are shown for overall survival **E.** and recurrence-free survival **F.**

The minimum *P*-value method for overall survival (OS) was used to determine H-scores of 162 and 80 as cutoff values for CD73 and A2AR, respectively. Using these cutoffs, 66 (10.3%) and 316 (49.2%) cases were classified as high CD73 expression and high A2AR expression, respectively. Patients were followed up for a median of 3.6 years (IQR, 1.9–5.8). There were 176 deaths from any cause and 260 recurrence-free survival (RFS) events. The estimated median survival time (MST) for OS was 11.8 years (95% CI, 9.4–not reached [NR]). Patients with NSCLC and high CD73 expression had a significantly worse OS (*P* = 0.0018) and RFS (*P* < 0.0001) than did patients with low CD73 expression (Figure 3A and 3B). The MSTs of CD73-high patients were 5.8 years (95% CI, 3.6–9.1) for OS and 2.0 years (95% CI, 1.0–4.0) for RFS, whereas those of CD73-low patients were 11.8 years (95% CI, 11.5–NR) and 9.5 years (6.5–NR), respectively. In contrast, patients with high A2AR expression had a significantly better OS (*P* = 0.00015) and RFS (*P* = 0.00012) than patients with low A2AR expression did (Figure 3C and 3D). The survival outcomes were extremely poor in patients with high CD73 and low A2AR expression, and patients with low CD73 and high A2AR expression showed the most favorable RFS outcomes (Figure 3E and 3F). A multivariate Cox regression analysis indicated that high CD73 expression was an independent unfavorable predictor for OS (hazard ratio [HR], 2.18; 95% CI, 1.38–3.46), whereas high A2AR expression was an independent favorable predictor (HR, 0.70; 95% CI, 0.50–0.98). Age, histology (nonadenocarcinoma nonsquamous histology vs. adenocarcinoma), and pathological stage were also independently associated with a poor prognosis (Table 2). For RFS, high CD73 expression (HR, 2.05; 95% CI, 1.42–2.95) and high A2AR expression (HR, 0.74; 95% CI, 0.56–0.97) were independent unfavorable and favorable prognostic factors, respectively, as well as pathological staging (Table 2).

**Table 2:** Results of multivariate Cox proportional hazards model analyses for overall survival and recurrence-free survival in patients with non-small-cell lung cancer or adenocarcinoma

Variable	Overall survival			Recurrence-free survival		
	Adjusted HR	95% CI	*P* value	Adjusted HR	95% CI	*P* value
**Non-small-cell lung cancer**						
Age (per additional year)	1.04	1.02–1.05	0.00016	1.01	0.99–1.02	0.24
Sex (male vs. female)	1.59	0.90–2.79	0.11	1.32	0.87–2.00	0.20
Smoking status (ever vs. never)	1.46	0.78–2.72	0.23	1.13	0.71–1.78	0.61
Histology (squamous vs. adenocarcinoma)	1.01	0.68–1.50	0.94	1.07	0.77–1.49	0.68
(others vs. adenocarcinoma)	1.82	1.11–2.98	0.018	1.36	0.88–2.10	0.17
Pathological stage (per additional stage)	2.18	1.80–2.64	<0.0001	2.34	1.99–2.74	<0.0001
Adjuvant chemotherapy (yes vs. no)	0.82	0.58–1.16	0.27	1.00	0.76–1.33	0.99
CD73 expression (high vs. low)	2.18	1.38–3.46	0.00089	2.05	1.42–2.95	0.00011
A2AR expression (high vs. low)	0.70	0.50–0.98	0.040	0.74	0.56–0.97	0.031
**Adenocarcinoma**						
Age (per additional year)	1.06	1.03–1.09	<0.0001	1.02	1.00–1.04	0.072
Sex (male vs. female)	0.72	0.34–1.52	0.39	0.68	0.38–1.19	0.18
Smoking status (ever vs. never)	3.22	1.43–7.27	0.0048	1.78	0.98–3.25	0.059
Pathological stage (per additional stage)	2.64	1.98–3.53	<0.0001	2.96	2.35–3.74	<0.0001
Adjuvant chemotherapy (yes vs. no)	0.83	0.49–1.42	0.50	0.79	0.53–1.19	0.26
Mutant EGFR expression (positive vs. negative)	1.07	0.61–1.90	0.81	1.32	0.85–2.04	0.22
TTF-1 positivity (positive vs. negative)	0.66	0.38–1.15	0.14	0.54	0.33–0.89	0.015
Subtypes (acinar vs. AIS/MIA/lepidic)				4.04	1.52–10.74	0.0052
(papillary vs. AIS/MIA/lepidic)				3.87	1.51–9.88	0.0047
(micropapillary vs. AIS/MIA/lepidic)				5.19	1.49–18.05	0.0096
(solid vs. AIS/MIA/lepidic)				3.37	1.21–9.38	0.020
(invasive mucinous vs. AIS/MIA/lepidic)				4.60	1.40–15.09	0.012
(colloid or enteric vs. AIS/MIA/lepidic)				42.77	4.48–408.70	0.0011
CD73 expression (high vs. low)	2.79	1.66–4.68	0.00011	2.27	1.47–3.51	0.00024
A2AR expression (high vs. low)	0.64	0.40–1.02	0.061	0.70	0.49–1.01	0.053

Abbreviations: HR, hazard ratio; CI, confidence interval; EGFR, epidermal growth factor receptor; TTF-1, thyroid transcription factor-1, AIS, adenocarcinoma in situ; MIA, minimally invasive adenocarcinoma

### CD73 and A2AR protein expression in adenocarcinoma of the lung

**Figure 4:**
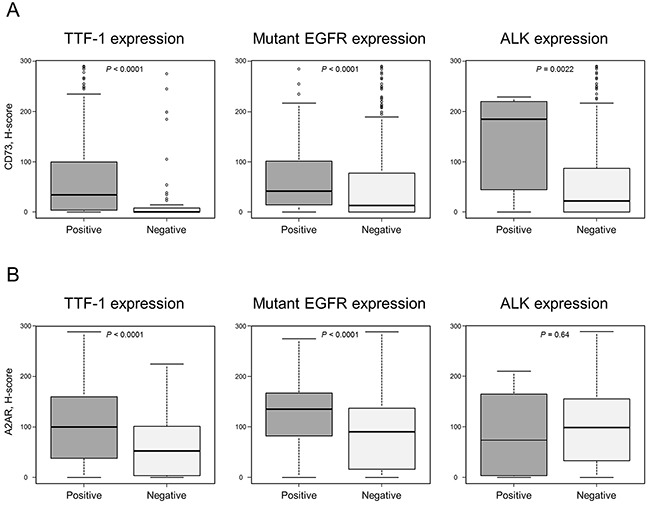
CD73 and A2AR expression levels in adenocarcinomas CD73 A. and A2AR B. expression levels are plotted according to TTF-1 positivity, mutant EGFR expression, and ALK positivity. Each box plot shows the median and interquartile range (top and bottom borders of the box). The whiskers above and below each box represent 1.5× of the interquartile range.

Next we performed clinicopathological and survival analyses of CD73 and A2AR expression in adenocarcinoma alone, as both proteins are prominently expressed in this tumor type. Among 422 adenocarcinomas, the most prevalent subtype was papillary (*N* = 181, 42.9%), followed by acinar (*N* = 76, 18.0%), solid (*N* = 51, 12.1%), lepidic (*N* = 49, 11.6%), adenocarcinoma in situ (*N* = 19, 4.5%), invasive mucinous (*N* = 18, 4.3%), minimally invasive adenocarcinoma (*N* = 15, 3.6%), micropapillary (*N* = 11, 2.6%), colloid (*N* = 1, 0.2%), and enteric (*N* = 1, 0.2%). Positive thyroid transcription factor-1 (TTF-1) expression was observed in 358 (85.0%) tumors (TTF-1 was not evaluable in one case because of core loss). Mutant epidermal growth factor receptor (EGFR) protein expression was observed in 119 (28.2%) patients, and positive anaplastic lymphoma kinase (ALK) expression was detected in 10 (2.4%) patients. CD73 and A2AR expression levels differed significantly according to histological subtype; CD73 expression levels were significantly higher in lepidic, acinar, papillary, and solid vs. invasive mucinous tumors, whereas A2AR expression levels were significantly higher in lepidic vs. acinar, solid, and invasive mucinous tumors (Supplementary Figure 2). Of note, significantly higher CD73 and A2AR expression was observed in TTF-1-positive tumors than in negative tumors, and in mutant EGFR-positive tumors than in negative tumors (Figure 4A and 4B). In addition, ALK-positive tumors also exhibited significantly higher CD73 expression levels than ALK-negative tumors did (Figure 4A). There were no significant differences in CD73/A2AR expression levels according to disease stage and, in contrast to the entire NSCLC cohort, A2AR expression levels did not differ significantly according to the intensity of immune cell infiltration (Supplementary Figure 1B).

### Survival analysis of CD73 and A2AR expression in adenocarcinoma of the lung

**Figure 5:**
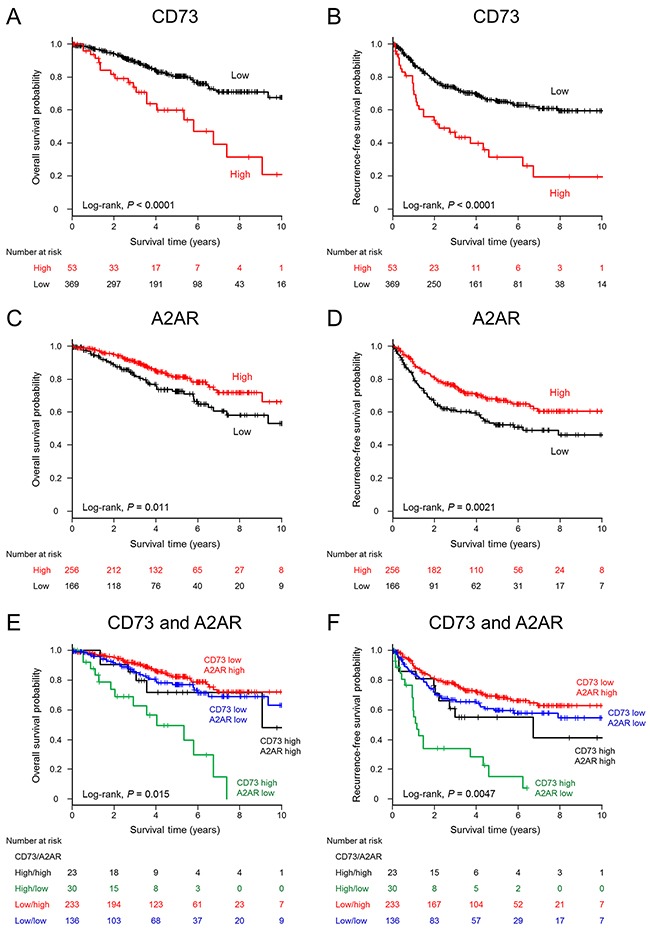
Kaplan–Meier estimates of overall survival and recurrence-free survival (years) stratified according to CD73 or A2AR expression levels in patients with lung adenocarcinoma Patients with adenocarcinoma and high CD73 expression had significantly worse overall survival **A.** and recurrence-free survival **B.** outcomes than did those with low CD73 expression. Adenocarcinoma patients harboring high A2AR expression had significantly better survival outcomes for overall survival **C.** and recurrence-free survival **D.** than did those with low A2AR expression. Survival estimations of adenocarcinoma patients stratified by combined tumor CD73 and A2AR expression status are shown for overall survival **E.** and recurrence-free survival **F.**

In this cohort, 89 deaths of any cause and 145 RFS events were reported. Similar to the results from the NSCLC cohort, significantly worse OS and RFS were observed among patients with high CD73 expression vs. low expression (both *P* < 0.0001, Figure 5A and 5B). The MSTs of OS were 5.8 years (95% CI, 3.6–9.1) for CD73-high adenocarcinomas and NR (95% CI, 11.5–NR) for CD73-low adenocarcinomas; the corresponding MSTs of RFS were 2.2 years (95% CI, 1.1–4.6) and NR (95% CI, 11.0–NR), respectively. In contrast, significantly better OS and RFS were observed in patients with high A2AR expression than in those with low A2AR expression (*P* = 0.011 for OS and *P* = 0.0021 for RFS, Figure 5C and 5D). The survival outcomes were very significantly poor in patients with high CD73 and low A2AR expression, as with the case of the entire NSCLC cohort (Figure 5E and 5F). As shown in Table 2, high CD73 expression was independently associated with a poor OS prognosis (HR, 2.79; 95% CI, 1.66–4.68). Age, smoking history, and pathological stage were also independent poor prognostic factors, whereas high A2AR expression was a borderline-significant favorable prognostic factor (HR, 0.64; 95% CI, 0.40–1.02). For RFS, high CD73 expression (HR, 2.27; 95% CI, 1.47–3.51) was an independent unfavorable prognostic marker, after adjusting for age, sex, smoking status, stage, adjuvant chemotherapy, mutant EGFR expression, TTF-1 expression, and adenocarcinoma subtypes (Table 2).

### Comparison of CD73 and A2AR protein expression in primary tumors and corresponding regional lymph node metastases

We comparatively analyzed the CD73 and A2AR expression profiles of primary tumors and synchronous metastatic lesions within regional lymph nodes to evaluate heterogeneity. Of 163 cases with lymph node metastases, 133 paired specimens were available for analysis. No significant difference in CD73 expression levels was observed between primary tumors and lymph node metastases (*P* = 0.14, Supplementary Figure 3), although the kappa statistics indicated poor agreement for positivity (kappa = 0.34; 95% CI, 0.11–0.57, Table 3) and a concordance rate of 80.5% (107 of 133). In contrast, significantly higher A2AR expression levels were observed in metastatic lesions vs. primary tumors (*P* < 0.0001, Supplementary Figure 3), and the agreement regarding positivity between primary and corresponding metastatic lesions was poorer than that observed for CD73 (kappa = 0.20; 95% CI, 0.04–0.36) with a concordance rate of 58.6% (78 of 133, Table 4).

**Table 3:** Agreement in the CD73 expression profiles between primary tumors and metastatic regional lymph nodes

Primary tumors	Lymph node metastases		Total
	High	Low	
High	11 (42.3%)	11 (10.3%)	22
Low	15 (57.7%)	96 (89.7%)	111
Total	26	107	133

Kappa coefficient of agreement, 0.34 (95% confidence interval, 0.11–0.57).

**Table 4:** Agreement in the A2AR expression profiles between primary tumors and metastatic regional lymph nodes

Primary tumors	Lymph node metastases		Total
	High	Low	
High	46 (52.3%)	13 (28.9%)	59
Low	42 (47.7%)	32 (71.1%)	74
Total	88	45	133

Kappa coefficient of agreement, 0.20 (95% confidence interval, 0.04–0.36).

## DISCUSSION

Given the widespread use of immune checkpoint inhibitors in patients with NSCLC, increasing attention has been paid to other molecules that might be involved in tumor immunoescape. In this study, we investigated the expression profiles of CD73 and A2AR in tumor cells and found that both proteins were more strongly expressed in adenocarcinoma, particularly TTF-1-positive and *EGFR*-mutant adenocarcinomas. To our knowledge, this study is the first to demonstrate the clinicopathological and prognostic implications of CD73 and A2AR in NSCLC. Remarkably, we found that CD73 and A2AR had independent and opposing prognostic impacts.

In contrast to intracellular adenosine, which is involved in energy and nucleic acid metabolism, extracellular adenosine has potent immunosuppressive effects and is detected at high levels within the tumor microenvironment [12, 24]. Extracellular adenosine triphosphate (ATP) derived from damaged or dying cells is considered a source of extracellular adenosine; ATP is converted to AMP via the intermediate adenosine diphosphate (ADP) by the ectoenzyme CD39, and AMP is further converted to adenosine by CD73 [6, 25]. CD73 is expressed on lymphocytes, endothelial and epithelial cells, and several types of cancer cells [25]. CD73 is considered a crucial checkpoint in this extracellular adenosine pathway [25], and CD73-generated adenosine binds to four different types of G-protein-coupled adenosine receptors. Stimulation of the A2AR on immune cells such as T cells, NK cells, NKT cells, and dendritic cells inhibits the activities of these cells [26], thus promoting an immunosuppressive tumor microenvironment [10–12]. Anti-CD73/A2AR cancer therapy has been identified as a potential novel therapeutic modality. Interestingly, functions of CD73 and A2AR within the tumor microenvironment have recently been reported not to be redundant, and co-inhibition of these interdependent pathways showed significantly greater anti-tumor efficacy than the single pathway-inhibitions [27]. In addition, combination treatments with anti-CD73/A2AR therapy and anti-PD-1 or anti-CTLA-4 therapy were found to be synergistic and promising therapeutic options [28–30]. The present study demonstrated that CD73 expression was more frequently and strongly observed in mutant EGFR-positive or ALK-positive tumors. Patients with gene alterations such as *EGFR* mutations and *ALK* translocations are considered less likely to respond to PD-1/PD-L1 inhibitors because of the relatively low frequency of PD-L1 expression and lack of effector T-cell trafficking into the tumor microenvironment [31]; this characteristic is probably attributable to the relatively low mutation burden, resulting in reduced generation of neopeptides that could be recognized by T-cells. Thus, the results of the present study suggest that CD73/A2AR-targeted therapeutics might be more effective in patients who would benefit less from anti-PD-1/PD-L1 therapy, because CD73 expression by tumor cells appears to serve as a biomarker of responses to therapies targeting the adenosine pathway [21, 29, 32].

In the present study, CD73 exhibited a remarkably worse prognostic value, which was somewhat expected from similar results observed in other cancers [17–21] and from the fact that CD73 has a wide range of oncogenic property in addition to immunosuppressive function [11, 13–16]. Moreover, the unfavorable prognostic impact of tumor CD73 expression was further enhanced when combined with low tumor A2AR expression. However, the reason underlying the survival benefit of high tumor A2AR expression is unclear as the functions of A2AR have mainly been studied in immune cells and in the context of the tumor microenvironment; specifically, A2AR expression on immune cells has been broadly considered to be immunosuppressive. In addition, only a few reports have investigated the function of A2AR in tumor cells. For example, A2AR activation was shown to decrease cell viability and induce cell death in a study using the pheochromocytoma cell line PC12 [33]. Conversely, knockdown of A2AR was found to decrease the growth of a lung cancer cell line, H1975 [34], and A2AR antagonists induced apoptotic cell death in PC9 and A549 lung cancer cell lines [9]. However, selective A2AR agonists had no effect on H1975 cell viability [34] and A2A blockade was suggested to act on host immune cells but not on tumor cells expressing A2AR [32]. The complex signaling pathway downstream to A2AR in tumor cells should be investigated to provide a mechanistic explanation of the different effects of A2AR.

To examine whether CD73 and A2AR expression heterogeneity has any importance with regard to tumor behavior during progression, we assessed the concordance of CD73 and A2AR expression levels between paired primary and metastatic specimens in this study. CD73 expression was generally concordant between primary and metastatic sites, whereas A2AR expression was significantly higher in metastatic lymph nodes than in primary tumors. We do not have an exact biological explanation for this finding. One possible explanation might be the reflection of an adaptive induction of A2AR via interactions between immune cells and tumor cells in the lymph nodes. However, considering our observation of a negative association between A2AR expression in primary tumors and the presence of tumor-infiltrating immune cells in entire NSCLC cohort, A2AR induction by infiltrating or proximal immune cells may not be plausible. In contrast to the presence of several rational explanations for CD73 expression by cancer cells [25, 27, 35–38], few explanations exist for A2AR expression and additional studies are needed.

The present study had several limitations. First, the retrospective nature of this study should be noted. To decrease potential biases, however, we collected large numbers of specimens from two institutions and investigated two survival outcomes, OS and RFS. Second, not all tumors were tested for *EGFR* mutations using standard PCR-based assays; only 268 tumors underwent standard analysis at commercial clinical laboratories (SRL in Tokyo [cycleave method], LSI Medience in Tokyo [peptide nucleic acid-locked nucleic acid PCR clamp method or the cobas EGFR assay], or BML, Inc. in Tokyo [PCR invader method]) as part of clinical practice. In the present study, the sensitivity, specificity, positive-predictive value, and negative-predictive value for mutant EGFR specific IHC were 76.3%, 94.3%, 84.1%, and 91.0%, respectively, when *EGFR* mutations (exon 19 deletions and L858R mutation in exon 21) identified via PCR-based assays were used as references. When only patients with adenocarcinoma who had been tested for *EGFR* alterations using standard methods (*N* = 186) were investigated, however, both the CD73 and A2AR expression levels remained significantly higher in *EGFR*-mutant tumors than in *EGFR*-wild type tumors (*P* < 0.0001 for CD73 and *P* = 0.0010 for A2AR). Finally, the results of assessment using the tissue microarrays (TMAs) might not completely reflect the true status of entire tumor specimens.

In conclusion, our data indicate that CD73 and A2AR are more commonly expressed in adenocarcinoma, particularly TTF-1-positive and mutant EGFR-positive adenocarcinoma. Despite these shared features, however, CD73 and A2AR expression could be used in an opposing manner to stratify patients with NSCLC and adenocarcinoma with respect to prognosis. Further studies are needed to confirm these results and provide an appropriate rationale for future CD73/A2AR-targeted therapeutics in the context of NSCLC management.

## MATERIALS AND METHODS

### Tumor specimen collection and tissue microarray construction

We collected a total of 653 resected NSCLC tumor specimens from 426 and 227 Japanese patients who underwent curative surgical treatment between January 1990 and December 2013 at Hamamatsu University Hospital (Japan) and between January 2006 and April 2014 at Seirei Mikatahara General Hospital (Japan), respectively. Written informed consent to use these specimens for medical research was obtained from all patients. This study was approved by the Institutional Review Boards of Hamamatsu University School of Medicine and Seirei Mikatahara General Hospital, and was conducted according to the principles laid down in the Helsinki Declaration. Clinical and pathological data were retrospectively obtained from a review of the patients’ medical records. Three board-certified pathologists histologically re-classified the tumors according to the 2015 World Health Organization Classification of Tumors of the Lung, Pleura, Thymus and Heart [39]. Cores for TMAs were isolated from representative lung cancer tissues and validated to contain sufficient tumor tissue by reviewing hematoxylin and eosin (HE)-stained sections, as reported previously [40]. Each individual core had a diameter of 2 or 3 mm. We also constructed TMAs from metastatic regional lymph nodes. The metastatic lymph node most distal from the primary tumor was selected in cases with multiple metastatic lymph node stations [41].

### Immunohistochemistry procedures and interpretation

TMA blocks were used for immunohistochemistry (IHC) analyses. We evaluated the expression levels of CD73, A2AR, and other proteins such as TTF-1, mutant EGFR with the exon 19 deletion or L858R point mutation, and ALK. IHC procedures were performed as described previously [41]. Antibodies against CD73 (dilution 1:200, clone D7F9A; Cell Signaling Technology (CST), Danvers, MA, USA), A2AR (dilution 1:400, clone SA654; Enzo Life Sciences, Farmingdale, NY, USA), TTF-1 (dilution 1:100, clone 8G7G3/1; Dako, Glostrup, Denmark), mutant EGFR with the exon 19 deletion (E746-A750; dilution 1:100, clone 6B6; CST) or L858R point mutation (dilution 1:100, clone 43B2; CST), and ALK (dilution 1:50, clone 5A4; Abcam, Cambridge, UK) were used. For ALK staining, the intercalated antibody-enhanced polymer method was applied. YI and KY independently assessed the expression status of each protein in a blinded manner, and consensus was obtained for discrepant results. To assess CD73, A2AR, and TTF-1 expression, semiquantitative H-scores were calculated by multiplying the intensity score (0, absent; 1, weak; 2, moderate; 3, strong) by the percentage of stained cells (0–100%) to yield a value of 0–300. Only membranous CD73 staining was interpreted, whereas both membranous and cytoplasmic A2AR staining were assessed (Figure 1). Specimens with H-scores that met or exceeded the individual cutoffs for CD73 and A2AR were defined as “high.” Stromal lymphocytes that exhibited positive staining were used as positive internal controls. Regarding TTF-1, nuclear staining of tumor cells was considered “positive,” and tumors without any nuclear TTF-1 expression (H-score of 0) were considered “negative” [42]. Mutant-EGFR and ALK protein expression was evaluated as described in our previous report [41]. Briefly, when assessing mutant EGFR, specimens with no or faint staining in <10% of tumor cells were considered “negative”; all others were considered “positive.” When assessing ALK, specimens with moderate or strong cytoplasmic staining were considered “positive” [41]. The intensity of immune infiltration was assessed on HE-stained sections and scored from 0 to 3 as follows: 0, no immune infiltration; 1, mild stromal immune infiltration (occupying <50% of the stromal surface area); 2, moderate stromal immune infiltration (occupying ≥50% of the surface area); and 3, strong immune infiltration that obscures the tumor (Figure 1). Scores of 0 and 1 were considered to indicate “low” immune infiltration, whereas scores of 2 and 3 were considered to indicate “high” infiltration [41]. All slides were analyzed using a bright-field microscope (Leica DMD108; Leica Microsystems, Wetzlar, Germany).

### Statistical analysis

The statistical analyses were performed using R software (R Foundation for Statistical Computing, Vienna, Austria, version 3.2.2). The cutoff values for CD73 and A2AR expression were determined using the minimum *P*-value method for OS. The Mann–Whitney *U* test or Kruskal–Wallis test was used for continuous variables, and *P* values in multiple comparisons were adjusted according to the method of Holm. Fisher's exact test was used to assess the association between CD73 and A2AR positivity defined by the above determined cutoff values. OS was defined as the interval between the date of surgery and date of death from any cause or last contact. RFS was defined as the interval from the date of surgery to the date of recurrence diagnosis, death from any cause, or last contact. Survival analyses were based on Kaplan–Meier estimations, and the log-rank test was used to analyze differences in survival durations. Cox proportional hazard regression models were fitted to determine the impacts of risk factors on OS and RFS after adjusting for disease and demographic covariates. The extents of similarities and differences in CD73 and A2AR expression levels between primary tumors and corresponding metastatic lymph nodes were analyzed using kappa statistics and the Wilcoxon rank-sum test, respectively. All statistical tests were two-sided, and *P* values <0.05 were considered statistically significant.

## SUPPLEMENTARY MATERIALS FIGURES


