# Supplementary material for: Prognostic impact of CD73 and A2A adenosine receptor expression in non-small-cell lung cancer
Source: Oncotarget. 2017 Jan 2;8(5):8738–51. doi: 10.18632/oncotarget.14434 (PMC5352437; doi:10.18632/oncotarget.14434)
Supplement: Supplementary file 1 [file oncotarget-08-8738-s001.pdf]

Prognostic impact of CD73 and A2A adenosine receptor expression in non-small-cell lung cancer

SUPPLEMENTARY FIGURES

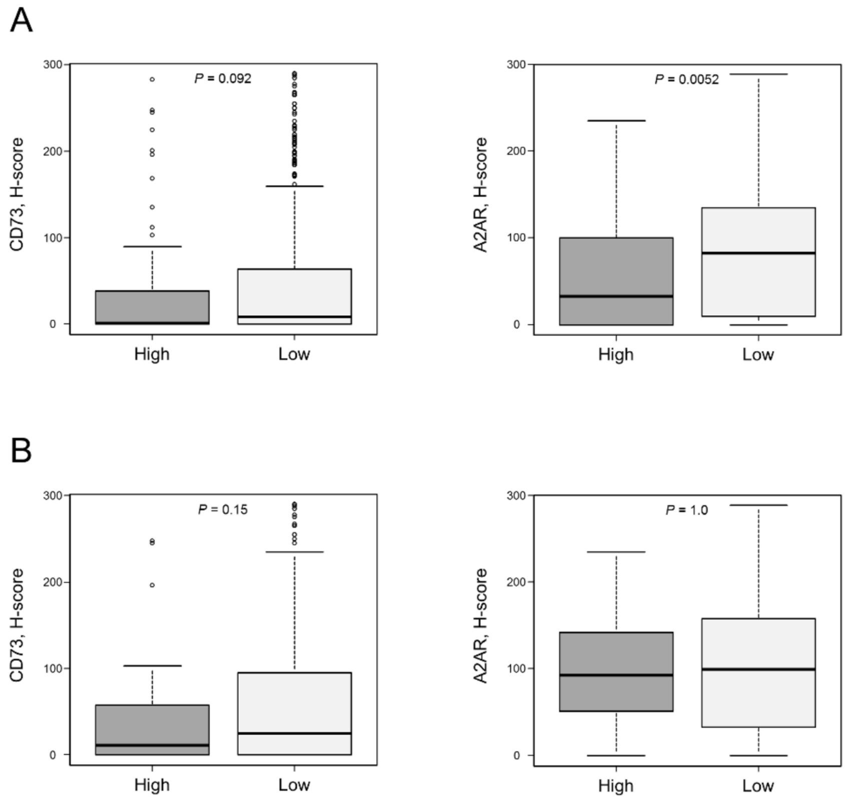

Supplementary Figure 1: CD73 and A2AR expression levels according to the intensity of immune infiltration. CD73 and A2AR expression levels in the entire non-small-cell lung cancer cohort **A.** and in adenocarcinomas **B.** are plotted. Each box plot indicates the median and interquartile range (top and bottom borders of the box). The whiskers above and below each box represent 1.5× of the interquartile range.

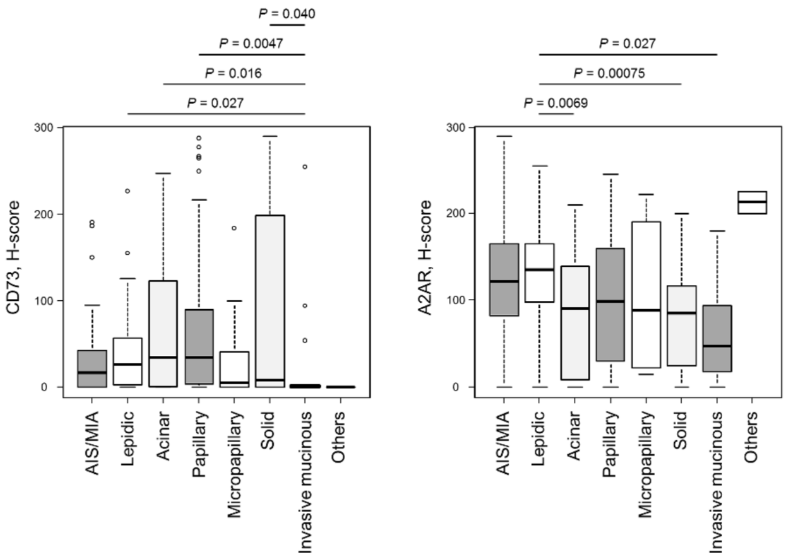

Supplementary Figure 2: Expression levels of CD73 and A2AR in adenocarcinoma of the lung according to the histological subtypes. Significant differences in CD73 (left) and A2AR (right) expression levels exist among the adenocarcinoma subtypes ($P = 0.0030$ for CD73 and $P < 0.0001$ for A2AR). Adjusted significant P values are shown. Each box plot shows the median and interquartile range (top and bottom borders of the box). The whiskers above and below each box represent 1.5× of the interquartile range. Others include colloid adenocarcinoma ($N = 1$) and enteric adenocarcinoma ($N = 1$). Abbreviations: AIS, adenocarcinoma in situ; MIA, minimally invasive adenocarcinoma.

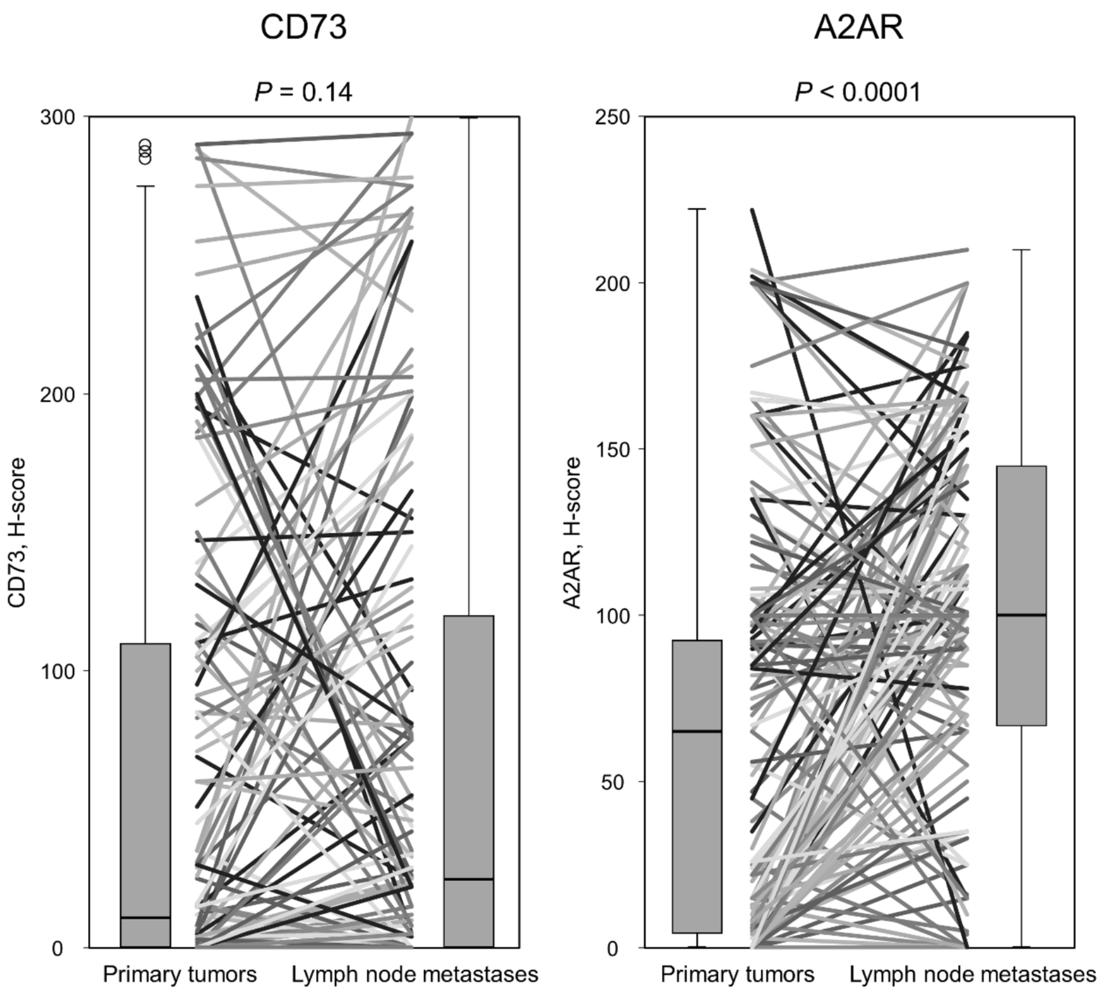

Supplementary Figure 3: Differences in CD73 and A2AR expression levels between primary tumors and corresponding metastatic lymph nodes. No significant difference in CD73 expression levels is detectable between primary and corresponding metastatic tumors (left). In contrast, A2AR expression levels are significantly higher in metastatic lymph nodes relative to corresponding primary tumors (right). Each box plot shows the median and interquartile range (top and bottom borders of the box). The whiskers above and below each box represent 1.5× of the interquartile range.
